# Rare severe hypofibrinogenemia induced by tissue plasminogen activator in stroke patients: Case report

**DOI:** 10.1097/MD.0000000000024978

**Published:** 2021-03-05

**Authors:** Xuming Huang, Liming Cao

**Affiliations:** a Department of Gastroenterology, Shenzhen Baoan Shiyan People's Hospital; b Shenzhen Baoan People's Hospital (group) The Second People's Hospital; c Department of Neurology, Shenzhen University First Affiliated Hospital; d Department of Neurology, The 3rd Affiliated Hospital of Shenzhen University, Shenzhen, China.

**Keywords:** case report, cerebral hemorrhage, hypofibrinogenemia, intravenous thrombolysis, recombinant tissue plasminogen activator

## Abstract

**Rationale:**

Severe hypofibrinogenemia after intravenous thrombolysis (IVT) with recombinant tissue plasminogen activator (rt-PA) is rare and easily overlooked, but hypofibrinogenemia increases the risk of major bleeding. However, it is unclear when hypofibrinogenemia reaches the peak and when hypofibrinogenemia is resolved.

**Patient concerns:**

Case 1 was of a 66-year-old man who was hospitalized due to sudden onset of vague speech and right hemiplegia for 4 hours. Case 2 was of an 84-year-old woman who was hospitalized for sudden onset of left hemiplegia and vague speech for 4 hours. In case 1, fibrinogen levels decreased from normal values to <0.25 g/L within 4.5 hours after commencing IVT and returned to normal at 35 hours later. In case 2, fibrinogen levels decreased from 1.1 to <0.25 g/L within 2 hours after commencing IVT and normalized 36.5 hours later.

**Diagnoses:**

Both patients were diagnosed with rt-PA-related hypofibrinogenemia.

**Interventions:**

No antiplatelet or symptomatic treatment was administered during the period of hypofibrinogenemia.

**Outcomes:**

Fibrinogen levels gradually recovered. In case 1, the patient did not experience cerebral hemorrhage during hypofibrinogenemia. His symptoms improved significantly within 1 week. In case 2, repeat computed tomography revealed minor cerebral hemorrhage, but no deterioration in her condition was noted until she was discharged.

**Lessons:**

Rapid, severe, and prolonged hypofibrinogenemia may occur after IVT with rt-PA, which may increase the risk of massive hemorrhage and affect the related therapy. Prompt diagnosis of hypofibrinogenemia is important for preventing complications. We recommend checking the fibrinogen levels routinely after IVT. Fibrinogen replacement therapy and platelet transfusion are the main management routes for rt-PA-related symptomatic intracranial hemorrhage.

## 1 Introduction

Intravenous thrombolysis (IVT) with recombinant tissue plasminogen activator (rt-PA) is the preferred treatment for acute ischemic stroke (AIS) within the 4.5-hour window, but rt-PA-related hypofibrinogenemia increases the risk of major bleeding, such as symptomatic intracranial hemorrhage (sICH).^[1]^ sICH occurs in 5.6% of AIS patients on intravenous rt-PA.^[2]^ Moreover, hypofibrinogenemia following rt-PA administration occurs in 13% of AIS patients,^[3]^ and severe hypofibrinogenemia is observed during IVT with rt-PA in nearly 5% of AIS cases.^[4]^ Fibrinogen assessment could be a rapid, inexpensive tool to identify patients at a high risk of bleeding.^[5]^ Early detection of hypofibrinogenemia after IVT is important for the prevention of major bleeding, but it is unclear when hypofibrinogenemia reaches the peak and when hypofibrinogenemia is resolved. Herein, we present 2 cases of severe rt-PA-related hypofibrinogenemia to clarify some points in its management.

## 2 Case presentation

### 2.1 Case 1

**Figure 1:**
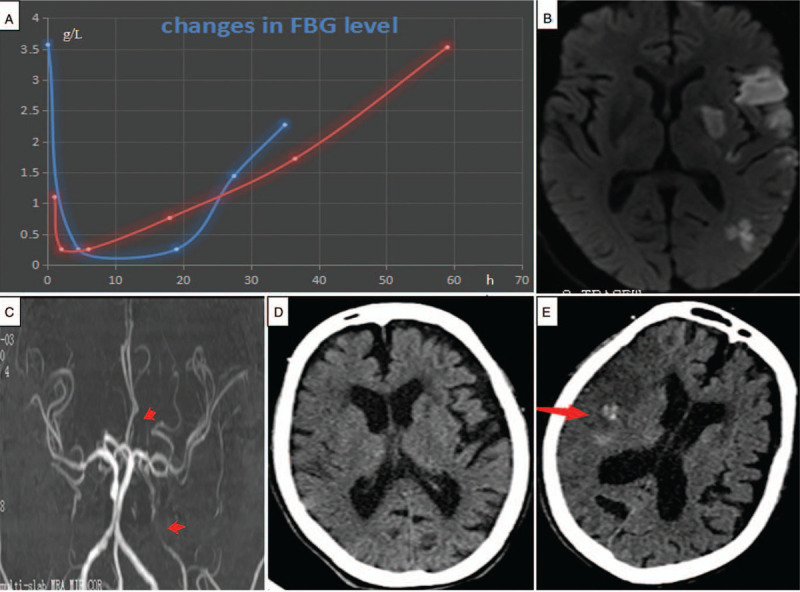
Changes in fibrinogen levels and neuroimaging results. (A) Changes in fibrinogen (FBG) levels in patient 1 (blue line) and patient 2 (red line) after intravenous thrombolysis (IVT) with recombinant tissue plasminogen activator (rt-PA). FBG levels sharply decreased immediately after IVT, and the peak status was sustained for hours, followed by a slight increase. Brain magnetic resonance (MR) diffusion-weighted imaging (B) shows multiple abnormal signals in the blood supply area of the left middle cerebral artery (MCA), suggesting acute watershed infarction in patient 1. Brain magnetic resonance angiography (C) shows severe stenosis of the left internal carotid artery (arrow) and signs of recanalization of the left MCA in patient 1. (D) Brain computed tomography (CT) before IVT shows a few old cerebral infarctions in bilateral subcortex areas in patient 2. (E) Brain CT on the second day after IVT shows a minor hemorrhage (arrow) within the focus of the right frontal and temporal lobe cerebral infarctions in patient 2.

A 66-year-old man was hospitalized for sudden onset of vague speech and right hemiplegia for 4 hours in April 2018. He developed a sudden inability to walk, and his symptoms continued to worsen. He had hypertension and cerebral infarction 5 years prior to admission but without sequelae. He denied any history of infectious diseases, major trauma, blood transfusion, alcoholism, drug abuse, and familial disease. He had smoked 50 cigarettes per day for >20 years. Admission physical examination findings were as follows: blood pressure of 151/87 mm Hg, body weight of 64.9 kg, drowsiness, mixed aphasia, a right shallow nasolabial sulcus, choking cough after drinking, muscular power of grade 2/5 in the right limbs, and absence of other positive neurological physical signs. The National Institute of Health Stroke Scale (NIHSS) score was 11. An emergent brain computed tomography (CT) did not show any obvious abnormality. The patient was diagnosed with AIS without absolute contraindication, and received immediate IVT with 50-mg rt-PA (0.77 mg/kg in 1 hour). At approximately 1 hour after rt-PA administration, right limb weakness improved significantly. At 4.5 hours after commencing IVT, the fibrinogen level declined sharply by >93.3% (<0.25 g/L, Table 1), and such low hypofibrinogenemia lasted for at least 14.5 hours. Fibrinogen levels returned to normal at 35 hours after commencing IVT (Table 1, Fig. 1A). He had no gingival bleeding, skin ecchymosis, hemoptysis, and other hemorrhagic symptoms, and physical examination showed no signs of deterioration. No hemorrhage was found on repeat brain CT on the second day after IVT. On laboratory analysis, white blood cell count (15.18 × 10^9^/L), platelet count (373 × 10^9^/L), and neutrophil percentage (76.8%) were increased. Fasting blood glucose level (2.9 mmol/L) was reduced. Levels of total bilirubin (21.4 μmol/L), alkaline phosphatase (148 U/L), urea (3.1 mmol/L), total cholesterol (5.33 mmol/L), and lactate dehydrogenase (263 U/L) were increased, while the levels of C-reactive protein (CRP), glycosylated hemoglobin, and thyrotropin were normal. A chest radiograph revealed enlargement of the cardiac silhouette. Brain magnetic resonance imaging showed acute multiple watershed infarctions in the area supplied by the left middle cerebral artery (Fig. 1B and C). He was treated with antiplatelet drugs (35 hours after IVT), hypolipidemic drugs, and a drug that improves brain circulation (intravenous vinpocetine 30 mg/day). He was discharged within 1 week, and his symptoms improved upon discharge (NIHSS score decreased from 11 to 1).

**Table 1:** Blood coagulation function before and after intravenous thrombolysis in patient 1 and 2.

	Patient 1					Patient 2						
Blood coagulation function marker	Before beginning IVT	4.5 h after beginning IVT	19 h after beginning IVT	27.5 h after beginning IVT	35 h after beginning IVT	Before beginning IVT	2 h after beginning IVT	6 h after beginning IVT	18 h after beginning IVT	36.5 h after beginning IVT	59 h after beginning IVT	Reference range
TT (s)	20.5	>150.0	51	22.4	20.9	28.2	73.6	56.6	26.8	17.5	18.4	14–20
PT (s)	10	32.7	24.9	11.3	11.1	15.8	23.4	19.4	14.0	13.9	12.5	10.5–14
INR	0.92	3.05	2.29	1.05	1.03	1.49	2.25	1.85	1.31	1.24	1.17	0.8–1.2
Fibrinogen, g/L	3.57	<0.25^∗^	<0.25^∗^	1.44	2.27	1.10	<0.25^∗^	<0.25^∗^	0.76	1.72	3.53	2–4
APTT (s)	20.6	45	38.7	19.8	19.2	58.2	105.4	56.1	41.5	41.7	33.5	21–34
Prothrombin activity (%)	192	18	26	118	127	48	27	34	60	73	74	88–120

### 2.2 Case 2

An 84-year-old woman was hospitalized because of a sudden onset of left hemiplegia and vague speech for nearly 4 hours. She denied having a history of any chronic disease, familial disease, or surgery. Her physical examination findings were as follows: blood pressure of 132/69 mm Hg, body weight of 40 kg, pinched look, clear consciousness, motor aphasia, muscular power of grade 2/5 in the left limb, and no other obvious positive neurological signs. The NIHSS score was 7. She underwent emergency head CT (Fig. 1D). Platelet count, red blood cell count, random blood glucose, and blood electrolytes showed no obvious abnormalities. Blood coagulation test results increased slightly but did not reach the level of contraindication. Her condition was diagnosed with AIS, and a total of 36 mg of rt-PA (0.9 mg/kg in 1 hour) was administered. The fibrinogen level declined sharply by >77.3% (<0.25 g/L, Table 1) at 2 hours after commencing IVT, and such low hypofibrinogenemia lasted for at least 4 hours. Fibrinogen levels returned to normal at 36.5 hours after rt-PA administration (Table 1, Fig. 1A). The serum creatinine, troponin I, and glycated hemoglobin levels were normal. The percentage of neutrophils (86.9%) was increased, and the hemoglobin concentration (85 g/L) was decreased, while the serum concentrations of B-type natriuretic peptide (3091 pg/mL), plasma D-dimers (1.85 mg/L), lactate dehydrogenase (272 U/L), CRP (92 mg/L), and homocysteine (19 μmol/L) were increased. A fecal occult blood test was weakly positive. One day after IVT, the muscle strength on the left limb was at a grade of 3/5 (NIHSS score, 6). Two days after IVT, brain CT showed a minor hematoma within the focus of the AIS (Fig. 1E). Three days after IVT, conventional chest CT showed an anomaly suspected to be aortic dissection (AD), and emergency CT angiography confirmed a thoracoabdominal AD (DeBakey type I). The patient's guardians refused surgical treatment for the AD; therefore, medications to control blood pressure and improve the metabolism of the brain cells and rehydration therapy were selected, but no antiplatelet treatment was provided. The patient left the hospital after 3 days, and changes in her condition were not significant at discharge. During the patient's follow-up visit 1.5 months later, her condition was the same as during discharge, but she was stable.

This study was approved by the ethics review board of the 3rd Affiliated Hospital of Shenzhen University (Approval no. 2019SZLH-LW-007). The patients provided written informed consent for the participation and publication of this report. They were satisfied with the treatment received and their recovery course.

## 3 Discussion

In this study, the patients had no significant liver function abnormalities, the fibrinogen level decreased quickly after IVT, no other reliable causes could be found, and hypofibrinogenemia may be attributed to hyperfibrinolysis secondary to IVT. The peak state of hypofibrinogenemia and resilience of fibrinogen level may be related to the patient's nutritional status, fibrinogen production capacity of the liver, and degree of consumption of fibrinogen. In case 2, malnutrition and marasmus led to low baseline fibrinogen levels; thus, more time was required for the normalization of the fibrinogen level. Our study showed that the peak normalization time after rt-PA administration is 2 to 4.5 hours, and the peak status can be maintained for at least 4 to 16.5 hours. The fibrinogen level decreased by >93.3% (3.32/3.57) and 77.3% (0.85/1.1) from the baseline in cases 1 and 2, respectively. In our study, the time taken to arrive at the peak value was shorter, the peak level was lower, the peak status lasted longer, and the recovery was slow than those reported previously.^[4]^

### 3.1 Significance of hypofibrinogenemia

During thrombolysis, rt-PA converts plasminogen into plasmin, and fibrin/fibrinogen is then degraded by plasmin to fibrin degradation products. In this phase, a significant amount of fibrinogen is consumed, resulting in hypofibrinogenemia. The fibrinolysis system is the most important anticoagulant system in the human body, and fibrinogen is a major coagulation factor.^[6]^ The low level of fibrinogen before/after IVT is a risk factor for hemorrhage.^[7,8]^ Fibrinogen levels of <2 g/L at 2 hours after IVT increases the risk of hematoma formation, and hypofibrinogenemia (<1.5 g/L) is associated with hematoma expansion in sICH.^[9]^ The intracerebral hemorrhage in case 2 may be associated with severe and prolonged hypofibrinogenemia. Hypofibrinogenemia is predictive of early parenchymal hematomas.^[9]^ Fibrinogen assessment may be a rapid, inexpensive, and widely available tool to aid in the identification of patients at a higher risk of bleeding.^[5]^ Early changes in fibrinogen levels may predict short-term efficacy of IVT in AIS. Specifically, relatively high fibrinogen levels at the first and fourth hours were positively associated with the short-term efficacy of IVT.^[10]^ However, in case 1, the patient's condition improved.

### 3.2 Causes of hypofibrinogenemia

The causes of hypofibrinogenemia include excessive consumption of fibrinogen, such as in disseminated intravascular coagulation; hyperfunction of fibrinolytic system, such as during IVT^[11]^; decreased fibrinogen synthesis, such as severe hepatitis and cirrhosis^[12]^; and drug-induced hypofibrinogenemia, such as that induced by allopurinol^[13]^ and adrenocorticotropic hormone.^[14]^

### 3.3 Monitoring of hypofibrinogenemia after rt-PA administration

Baseline fibrinogen levels of 3.35 ± 0.82 g/L decreased to 2.52 ± 0.83 g/L at 2 hours after the commencement of IVT in a large-sample study.^[15]^ We could not assess the fibrinogen level at any time, and we needed to know the peak time of hypofibrinogenemia and how long the peak will last, so that we can efficiently monitor hypofibrinogenemia. The fibrinogen levels decreased at 2 hours after rt-PA treatment and remained low after 24 hours.^[16]^ In another large-sample study,^[8]^ the fibrinogen level decreased to its peak value at 6 hours after rt-PA administration and did not return to its pre-thrombolysis level at 24 hours. At 5 hours, the plasma plasminogen level decreased by 57% after rt-PA administration.^[11]^ There is no report on the usual length of the peak.^[4]^ Considering the characteristics of the patients (elderly,^[17]^ dystrophy patients, patients who are at risk for hypofibrinogenemia, and patients with hemorrhage), clinicians can adjust the detection times appropriately. The Chinese guidelines^[18]^ for AIS recommends the use of antiplatelet drugs at 24 hours after rt-PA administration; however, this may increase the risk of excessive bleeding in patients with hypofibrinogenemia. rt-PA-related hypofibrinogenemia increases the risk of sICH after IVT. We conclude that the use of antiplatelet or anticoagulant therapy when the fibrinogen level does not return to normal increases the risk of bleeding. Antiplatelet or anticoagulant therapy may be safer after the fibrinogen levels have returned to normal.

### 3.4 Treatment of rt-PA-related sICH

Fibrinogen replacement therapy^[19]^ (e.g., fresh-frozen plasma [FFP] and cryoprecipitates) and platelet transfusion are the main management routes for rt-PA-related sICH. The effect of FFP on rt-PA-induced sICH may be rapid, and the FFP dose recommended by the British Hematological Standards Committee is 12 mL/kg.^[20]^ Cryoprecipitates are derived from FFP, which contains fibrinogen. Once rt-PA-related sICH is diagnosed, therapists may consider empirically importing 10 U of cryoprecipitate and administering more, as needed, to achieve a fibrinogen level ≥1.5 g/L.^[21]^ Transfusion of 6 to 8 U of platelets is also a routine recommendation for the treatment of rt-PA-related sICH.^[22]^

Such rapid, severe, and prolonged hypofibrinogenemia is rarely reported. Further large-scale studies will be needed to confirm our findings.

In conclusion, rapid, severe, and prolonged hypofibrinogenemia may occur after IVT, which may increase the risk of massive hemorrhage and affect the application of antiplatelet and anticoagulant therapy. Prompt diagnosis of hypofibrinogenemia after IVT is the key to preventing massive bleeding. Fibrinogen levels should be monitored within 2 hours after beginning IVT, and monitoring must be performed even after 24 to 36 hours after IVT. Therefore, we recommend checking the fibrinogen levels routinely before IVT and at 2, 6, 12, 24, and 36 hours after IVT. Fibrinogen replacement therapy (e.g., FFP and cryoprecipitates) and platelet therapy are the main management routes for rt-PA-related sICH.

## Acknowledgments

The authors would like to thank Editage (www.editage.com) for English language editing.

## Author contributions

**Conceptualization:** Liming Cao.

**Data curation:** Xuming Huang.

**Writing – original draft:** Liming Cao.

**Writing – review & editing:** Liming Cao.

## References

[1] Skeik N Gits CC Ehrenwald E. Fibrinogen level as a surrogate for the outcome of thrombolytic therapy using tissue plasminogen activator for acute lower extremity intravascular thrombosis. Vasc Endovascular Surg 2013;47:519–23. 23899656 10.1177/1538574413497107

[2] Seet RC Rabinstein AA. Symptomatic intracranial hemorrhage following intravenous thrombolysis for acute ischemic stroke: a critical review of case definitions. Cerebrovasc Dis 2012;34:106–14. 22868870 10.1159/000339675

[3] Smith K Fraser G Seder D. Hypofibrinogenemia and abnormal INR after treatment with systemic recombinant tissue plasminogen activator for acute ischemic stroke. Crit Care Med 2012;40:1–328.23213646

[4] Matrat A De Mazancourt P Derex L. Characterization of severe hypofibrinogenemia induced by alteplase in two patients thrombolysed for stroke. Thromb Res 2013;131:e45–8. 23199548 10.1016/j.thromres.2012.11.009

[5] Vandelli L Marietta M Gambini M. Fibrinogen decrease after intravenous thrombolysis in ischemic stroke patients is a risk factor for intracerebral hemorrhage. J Stroke Cerebrovasc Dis 2015;24:394–400. 25497721 10.1016/j.jstrokecerebrovasdis.2014.09.005

[6] Lowe GD Rumley A Mackie IJ. Plasma fibrinogen. Ann Clin Biochem 2004;41:430–40. 15588432 10.1258/0004563042466884

[7] Xu XH Li CS Wan T. Risk factors for hemorrhagic transformation following intravenous thrombolysis in acute cerebral infarction: a retrospective single center study. World Neurosurg 2017;101:155–60. 28185970 10.1016/j.wneu.2017.01.091

[8] Matosevic B Knoflach M Werner P. Fibrinogen degradation coagulopathy and bleeding complications after stroke thrombolysis. Neurology 2013;80:1216–24. 23486872 10.1212/WNL.0b013e3182897015

[9] Sun X Berthiller J Trouillas P. Early fibrinogen degradation coagulopathy: a predictive factor of parenchymal hematomas in cerebral rt-PA thrombolysis. J Neurol Sci 2015;351:109–14. 25783009 10.1016/j.jns.2015.02.048

[10] Lu T Xian W Liang J. Early changes in fibrinogen after administration of alteplase are associated with the short-term efficacy of thrombolysis. Medicine (Baltimore) 2018;97:3. 10.1097/MD.0000000000010241 PMC5895368 29595678

[11] Rao AK Pratt C Berke A. Thrombolysis in myocardial infarction (TIMI) trial—Phase I: hemorrhagic manifestations and changes in plasma fibrinogen and the fibrinolytic system in patients treated with recombinant tissue plasminogen activator and streptokinase. J Am Coll Cardiol 1988;11:1–1. 3121710 10.1016/0735-1097(88)90158-1

[12] Parker GW Finkel M Reynolds RD. Hypofibrinogenemia without fibrinolysis due to cirrhosis: report of a rare occurrence. Gastroenterology 1964;46:778–81.

[13] Yin Z Xu J Li Y. Transient hypofibrinogenemia due to allopurinol. Drug Des Devel Ther 2014;8:1231–3. 10.2147/DDDT.S66868 PMC4159221 25214766

[14] Kamei A Araya N Akasaka M. Hypofibrinogenemia caused by adrenocorticotropic hormone for infantile spasms: a case report. Brain Dev 2015;37:137–9. 24735983 10.1016/j.braindev.2014.03.004

[15] Yan S Zhang X Zhang R. Early fibrinogen depletion and symptomatic intracranial hemorrhage after reperfusion therapy. Stroke 2019;50:2716–21. 31394994 10.1161/STROKEAHA.119.025711

[16] Tanne D Macko RF Lin Y. Hemostatic activation and outcome after recombinant tissue plasminogen activator therapy for acute ischemic stroke. Stroke 2006;37:1798–804. 16763191 10.1161/01.STR.0000226897.43749.27

[17] Stangl K Laule M Tenckhoff B. Fibrinogen breakdown, long-lasting systemic fibrinolysis, and procoagulant activation during alteplase double-bolus regimen in acute myocardial infarction. Am J Cardiol 1998;81:841–7. 9555772 10.1016/s0002-9149(98)00018-6

[18] Neurology Society of Chinese Medical Association; Cerebrovascular Diseases Group, Neurology Society, Chinese Medical Association. Chinese guidelines for the diagnosis and treatment of acute ischemic stroke 2018. Chin J Neurol 2018;51:666–82. [Chinese].

[19] Besser MW MacDonald SG. Acquired hypofibrinogenemia: current perspectives. J Blood Med 2016;7:217–25. 27713652 10.2147/JBM.S90693 PMC5045218

[20] Makris M Van Veen JJ Tait CR. Guideline on the management of bleeding in patients on antithrombotic agents. Br J Haematol 2013;160:35–46. 23116425 10.1111/bjh.12107

[21] Yaghi S Willey JZ Cucchiara B. Treatment and outcome of hemorrhagic transformation after intravenous alteplase in acuteischemic stroke: a scientific statement for healthcare professionals from the American Heart Association/American Stroke Association. Stroke 2017;48:e343–61. 29097489 10.1161/STR.0000000000000152

[22] Adams HP Brott TG Furlan AJ. Guidelines for thrombolytic therapy for acute stroke: a supplement to the guidelines for the management of patients with acute ischemic stroke. a statement for healthcare professionals from a Special Writing Group of the Stroke Council, American Heart Association. Stroke 1996;27:1711–8. 8784157

